# Technical realization of a sensorized neonatal intubation skill trainer for operators’ retraining and a pilot study for its validation

**DOI:** 10.1186/s13052-017-0435-z

**Published:** 2018-01-04

**Authors:** Davide Panizza, Rosa T. Scaramuzzo, Francesca Moscuzza, Ilaria Vannozzi, Massimiliano Ciantelli, Marzia Gentile, Ilaria Baldoli, Selene Tognarelli, Antonio Boldrini, Armando Cuttano

**Affiliations:** 1 Istituto di Scienze della Vita, Scuola Superiore Sant’Anna, Piazza Martiri della Libertà, 33, 56127 Pisa, Italy; 2 U.O. Neonatologia, Azienda Ospedaliero-Universitaria Pisana (AOUP), Pisa, Italy; 3 Centro di Formazione e Simulazione Neonatale “NINA”, U.O. Neonatologia, Azienda Ospedaliero-Universitaria Pisana (AOUP), Pisa, Italy; 4 Università di Pisa, Pisa, Italy; 5 The BioRobotics Institute, Scuola Superiore Sant’Anna, Pontedera, Italy

**Keywords:** Neonatal intensive & critical care, Medical Education & Training, Neonatology

## Abstract

**Background:**

In neonatal endotracheal intubation, excessive pressure on soft tissues during laryngoscopy can determine permanent injury. Low-fidelity skill trainers do not give valid feedback about this issue. This study describes the technical realization and validation of an active neonatal intubation skill trainer providing objective feedback.

**Methods:**

We studied expert health professionals’ performances in neonatal intubation, underlining chance for procedure retraining. We identified the most critical points in epiglottis and dental arches and fixed commercial force sensors on chosen points on a ©Laerdal *Neonatal Intubation Trainer*. Our skill trainer was set up as a grade 3 on Cormack and Lehane’s scale, i.e. a model of difficult intubation. An associated software provided real time sound feedback if pressure during laryngoscopy exceeded an established threshold. Pressure data were recorded in a database, for subsequent analysis with non-parametric statistical tests. We organized our study in two intubation sessions (5 attempts each one) for everyone of our participants, held 24 h apart. Between the two sessions, a debriefing phase took place. In addition, we gave our participants two interview, one at the beginning and one at the end of the study, to get information about our subjects and to have feedback about our design.

**Results:**

We obtained statistical significant differences between consecutive attempts, with evidence of learning trends. Pressure on critical points was significantly lower during the second session (*p* < 0.0001). Epiglottis’ sensor was the most stressed (*p* < 0.000001). We found a significant correlation between time spent for each attempt and pressures applied to the airways in the two sessions, more significant in the second one (shorter attempts with less pressure, r_s_ = 0.603).

**Conclusions:**

Our skill trainer represents a reliable model of difficult intubation. Our results show its potential to optimize procedures related to the control of trauma risk and to improve personnel retraining.

**Electronic supplementary material:**

The online version of this article (10.1186/s13052-017-0435-z) contains supplementary material, which is available to authorized users.

## Background

Neonatal endotracheal intubation (EI) is recommended for airway management in several clinical scenarios. It is complex and requires a great deal of clinical experience: it must be completed within 30 s [1] in order to minimize hypoxia and performed correctly to avoid complications. Several injuries may result from inaccurately performed intubation maneuvers: hypoxia, trachea or esophagus injuries [1], soft tissue damage with long term effects, e.g. dentition and palate anomalies [2, 3].

A proper knowledge of the airway anatomy is a key point for an effective execution of EI [4]. Cormack and Lehane scale allows a preventive evaluation of EI difficulty considering glottis view at direct laryngoscopy [5].

Simulation-based learning is a valid instrument for acquisition and maintenance of clinical and procedural skills in healthcare, as well as to focus on non-technical skills, i.e. team communication and crisis management and resolution [6, 7]. Its use is rapidly growing in neonatal clinical education [8], as it permits exercising clinical skills without any patient damage. Healthcare providers get the chance to improve their competence in invasive procedures like EI, analyzing their own mistakes or misunderstandings via structured debriefing.

There are several neonatal intubation manikin commercial models, all with a head-neck structure and airways, e.g. *Infant Delux Simulation Head (©Simulaids), Neonatal Intubation Trainer (©Laerdal), Infant Airway Management Trainer (©NascoLifeform)* and *AirsimBaby (©Trucorps).* However, they are passive instruments as do not provide any real-time feedback about the accuracy of the maneuver. The operator hence needs an expert tutorship during training, which can only give a qualitative opinion on performances. Moreover, hard plastic manikins do not appropriately resemble soft living tissue, requiring much more pressure for intubation tasks. Bishop et al. stated that ©Laerdal manikins’ intubation needs the application of an elevated average force, probably due to the materials used for some anatomical components, especially the tongue: stiff plastic needs a maximal pressure to obtain an initial deformation, and a significant pressure to complete the procedure [9]. The result justifies the opinion of those clinicians asserting that the manikin is more difficult to intubate than the human newborn.

In this study, in order to improve education programs we worked on the setup and clinical validation of an innovative active device, based on the sensorization of a commercial newborn EI skill trainer, allowing the operator to verify his own execution real time and through structured debriefing. In particular, our primary outcome measures were the pressure on critical points during the procedure and the time spent for the procedure itself. As a secondary outcome, we investigated the participants’ satisfaction towards the skill trainer and the simulation itself.

## Methods

### Assembly of skill trainer

We chose the commercial *©Laerdal Neonatal Intubation Trainer* for our study. This device is currently used for education and maintenance of intubation skills in health professionals. To obtain objective feedbacks during the intubation procedure, we fixed three commercial force sensors (FSR 400 short, *©Interlink Electronics*) [10, 11] with a small size and a sensitivity range of 0.2–20 Newton (N) on the skill trainer. On the basis of previous investigations [2, 3] and clinical evidence, we considered three major anatomical structures as critical points of damage during laryngoscopy: epiglottis, superior dental arch and inferior dental arch. We attached force sensors with Parafilm M© on the airway cast external face (Additional file 1: Figure S1A). Parafilm M© is a thermoplastic, self-sealing film that keeps moisture loss to a minimum and offers an excellent barrier protection to the contents of tubes, flasks and culture tubes. In addition, we used a soft base for the epiglottis sensor to obtain a better stabilization on the mannequin. We had previously tried to anchor sensors by simply placing glue on the internal side of the cast, but this solution did not guarantee sensor stability and glue corroded the plastic structures of the manikin [12]. Finally, we attached two metal layers at the basis of the neck to provide a signal of reciprocal positions of neck and head during intubation.

The idea for such an assembly comes from reported problems about skill trainer’s fidelity, particularly clinicians’ opinion about higher pressure exercised on skill trainer compared to clinical practice. In our opinion, a possible way to solve skill trainers’ lack of fidelity is considering them as difficult intubation models: in fact, in clinical practice, a difficult intubation often needs higher pressure. In our assembly, the use of Parafilm M© allowed us to increase skill trainer stiffness, in order to obtain a poor view of the glottis and consequently a difficult intubation model. We classified intubation difficulty as grade 3 on Cormack and Lehane’s scale [5]. Intubation difficulty grade was the same in all intubation attempts carried out by our participants.

In our opinion, offering skill training on a high-difficulty model allows the optimization of the procedure, achieving an automated execution of gentler gestures in easier clinical situations. Moreover, it encourages a deeper reflection on the procedure by the participants. Our opinion about skill trainer is shared by most of our participants, as demonstrated by the results of the second interview.

Our skill trainer is characterized by a double feedback: real time alarm sound informs operators of the inappropriate pressures reached during the procedure, while data recording permits post-session debriefing. Moreover, real time feedback allows a playful approach to the issue, which guarantees a serene and motivated participation in the sessions, according to simulation principles.

### Software development

We envisioned LabVIEW software with user-friendly graphic interface (GUI) for device control, including control of pressure, control of head position and timer (Additional file 1: Figure S1B).

The GUI related to force registration consists of a traffic light coding for each sensor. The software also provides a sound alarm, in order to give real time feedback for pressures above threshold during intubation attempts. Threshold values (i.e. 7 N for the epiglottis and 2 N for dental arch) were the mean pressures obtained by three particularly competent participants (expert performance). We opted for this solution because so far no data have been reported about pressure threshold values on airway soft tissues, to the best of our knowledge. The so obtained (and chosen) alarming pressure was quite low, but we gladly accepted it since our manikin model was conceived as a skill trainer for difficult intubation. The GUI related to head position shows a similar coding even through it has a different alarm sound used just for informing of head hyperextension, and no force threshold are required. Finally, in the last part of the GUI we included a chronometer supplied with an alarm sound (the first alarm is set 30 s after the beginning of the intubation attempt and then every 10 s, according to NRP guidelines, which suggest that an intubation attempt should not last over 30 s [13]).

Pressure values during the sessions are recorded in electronic database with 0.1 s intervals, and the LabVIEW software generates real time graphs, providing a view of procedure quality.

### Study design

We recruited 14 medical professionals (7 male, 7 female) from the Neonatology Department in Pisa University Hospital (Italy), all experienced in neonatal intensive care. They all took part in two intubation sessions (each participant carried out 5 intubation attempts for each intubation session for a total of 10 attempts each) held 24 h apart, with debriefing taking place between them. An intubation attempt started when the operator took the laryngoscope to execute the maneuver, and finished with the success of the procedure or its failure declared by the operator himself. The time taken by the procedure was that between these two points.

Since intubation is a maneuver executed singularly, we think an individual debriefing was a more suitable option for our study. After the first session, the debriefer collected personal impressions of each participant. Each participant had the chance to think about his intubation technique and his difficulty in the previous intubation attempts. After 24 h, before the second intubation session, the debriefer discussed these issues with the participant and managed to make him aware of the problem of damage in intubation and to describe our skill trainer as a model of difficult intubation to study this same problem.

Intubation attempts were conducted with a video-laryngoscope C-Mac Storz with n.1 blade, with a neonatal rigid uncuffed endotracheal tube (3.5 mm). We chose a video-laryngoscope to give our operators the chance of a video-assistance, considering the difficulty of intubation maneuver in our skill trainer. The use of video-assistance was suggested by the debriefer after the first intubation session.

All information was anonymously collected: time of procedure, pressure on critical points and number of above-threshold events.

All participants were delivered two surveys to fill out, one at the beginning and one at the end of the study, completely anonymous. The first interview had the purpose of characterizing our group: gender, years of experience in Neonatal Intensive Care Unit (NICU), number of intubations performed per year, intubation competence self-evaluation, opinion about any previously attended simulation courses and general opinion about simulation and its usefulness in clinical practice. The second interview evaluated the appreciation of study design, self-assessment of performances and asked to compare the experience in simulation with routine clinical practice.

Ethics Committee approval was not requested because our study did not involve either patients or animals.

### Statistics

For the data analysis, we used the following statistical test (non-parametric statistics):

1. Chi-square test: to compare categorical variables between two groups of paired data;
2. Mann-Whitney Test: for comparison between two groups of samples (quantitative variables) with independent data.
3. Wilcoxon Test: for comparison between two groups of samples (quantitative variables) with paired data;
4. Kruskar-Wallis Test: for the comparison of more than two groups (quantitative variables) with independent data;
5. Friedman Test – one way ANOVA: for comparison between more than two groups of samples (quantitative variables) with paired data;
6. Comparison test between two quantitative variables, with expression of the strength by the Spearman correlation coefficient

## Results

The description of participants who completed the study is reported in Table 1 - part A.

**Table 1:** Results of the two interviews given to all participants

(PART A) First interview: participants’ characteristics		
Number and gender	7 male, 7 female	
NICU work experience	15–25 years (mode > 20)	
Number of EI per year	5–15	
Self-assessment in EI skills (mean score)	8*	
Evaluation of previous simulation courses	9*	
Usefulness of simulation in EI learning	9*	
Usefulness of simulation-based retraining in EI	9*	
(PART B) Second interview: participants’ feedback		
FEATURE	JUDGEMENT	
Skill trainer	8*	
Skill trainer fidelity	7*	
Skill trainer mechanical rigidity	6*	
Participant difficulties in intubation procedure due to high stiffness property of the skill trainer	9 of 14 participants (64%)	
Skill trainer usefulness in difficult EI retraining	In 71% of the participants	8,6*
Study design	8,4*	
Overall performance evaluation	7,9*	
Performance improvement evaluation	8*	
Comparison with force for EI in clinical practice	6 participants (43%): Same force	
	2 participants (14%): Lower force	
	4 participants (29%): Higher force	
	1 participant: Higher force in 1st session, lower force in 2nd session	
	1 participant: Comparison depending on neonatal age and sedation	
Debriefing importance	7,7*	
Familiarization phase	Necessary for 9 participants (64%)	
EI performance with poor glottis view	Possible for 5 participants (35%)	
Epiglottis loading in EI	Necessary for 5 participants (35%)	
Mental phase by phase analysis of the EI maneuver	Important for 7 participants (50%)	9,1*
Skill trainer usefulness in EI post hoc analysis	7,8*	

*values are referred to an evaluation graded scale from 1 to 10. EI: endotracheal intubation

Our debriefing was successful in making the operators aware of the problem of damage in intubation maneuver. All of them shared the importance of this issue, and paid more attention to this in the second intubation session, as showed in our results. No one of the operators underlined problems in using video-laryngoscope instead of classic laryngoscope. However, nobody used video-assistance during intubation execution in the first session, preferring to use the instrument as a traditional laryngoscope. In addition, the debriefer suggested them to take advantage of video-assistance during the second intubation session, but most participants adopted direct laryngoscopy anyway.

The first step of our analysis focused on learning trends with respect to procedure time and pressure on critical points, testing differences between consecutive attempts with Friedman test (one-way ANOVA).

**Fig. 1:**
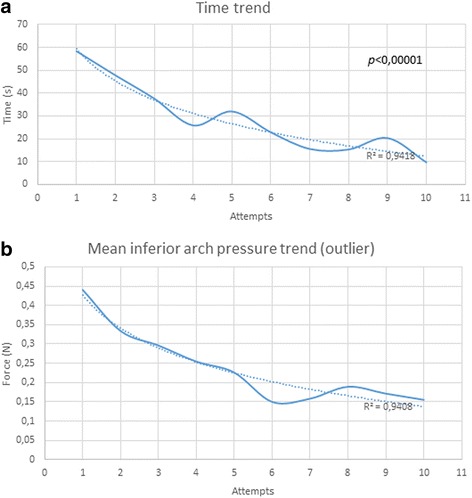
Learning trends during skill trainer sessions. **a** Time of procedure in two sessions. **b** Mean inferior arch pressure in two sessions

For each attempt, we assessed the mean procedure time values of all the operators considered together to establish learning trends (Additional file 2: Table S2). For the first session, we obtained a third grade polynomial function with R^2^ = 0.99, with significant differences (*p* < 0.00001) between consecutive attempts. In the second session, by excluding two outliers we obtained another third grade polynomial function with R^2^ = 0.99 with significant differences between consecutive attempts (*p* < 0.001). After considering the two sessions together, without excluding outliers, learning trends could be assimilated to a logarithmic function (R^2^ = 0.94, *p* < 0.00001 - Fig.1a).

We tested the hypothesis that statistical significant differences existed between force exerted on critical points. For this purpose, we used Kruskal Wallis test (one-way ANOVA).

To determine the learning trends based on force values on critical points, we considered for each attempt the mean force applied by each participant on force sensors (Additional file 3: Table S3).

On the epiglottis sensor, we obtained a stable trend for the first session (between 2.82 N and 3.17 N). In the second session, by excluding an outlier we observed a significant difference between consecutive attempts (*p* < 0.01). Considering both sessions together, we obtained a linear function (R^2^ = 0.82).

For superior dental arch sensor, considering both overall sessions, we obtained a third grade polynomial function (R^2^ = 0.83).

For the inferior dental arch sensor, excluding an outlier, the learning trend was represented by a logarithmic function both in the first session (R^2^ = 0.99) and considering two sessions together (R^2^ = 0.94) -Fig.1b.

**Fig.2:**
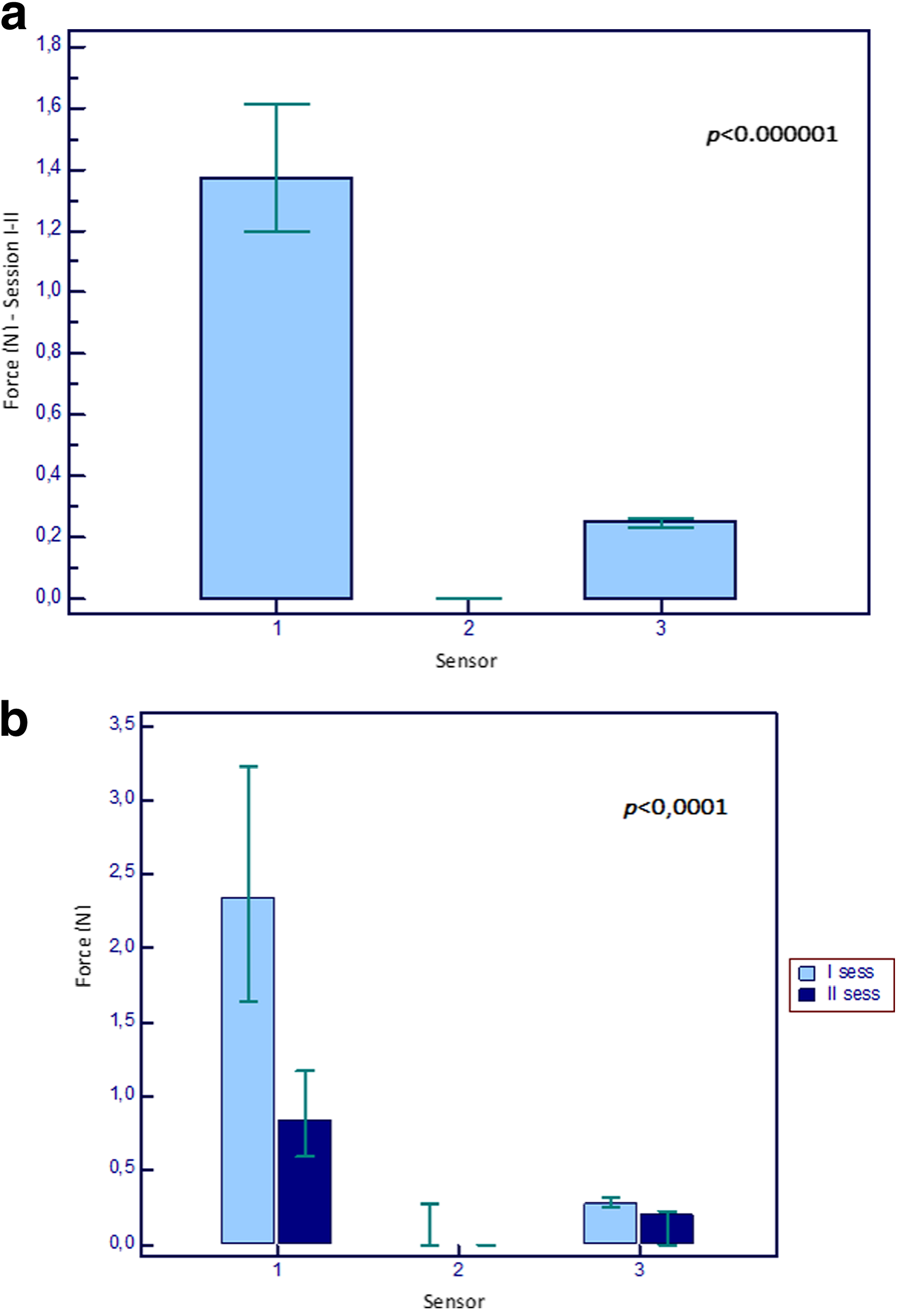
**a** Differential stress on three sensors in the two sessions. **b** Comparison of pressures exercised on three sensors between I and II session. 1.Epiglottis; 2. Superior arch; 3. Inferior arch

The epiglottis force sensor was the most stressed, followed by the sensor on the inferior dental arch and that on the superior arch (*p* < 0.000001) - Fig.2a. Using Wilcoxon rank test, we found a significant between-session difference for epiglottis (median 2.85 vs. 0.84) and inferior dental arch sensor (median 0.28 vs. 0.21), *p* < 0.0001 (Fig.2b).

We assessed if different age groups in our professional sample could exhibit differences in the considered variables using Kruskal-Wallis test.

We divided all participants in three groups (post hoc analysis): gr. A (<10 years of NICU work experience), gr. B (10–20 years of NICU work experience), gr. C (more than 20 years of NICU work experience). We did not find any significant differences between three groups for procedure time in any session. We evidenced minor pressures for group A on epiglottis (median gr. A 1.99 vs. gr. B 3.4 vs. gr. C 3.03) in the first session (*p* < 0.01) but not in the second one, and stronger pressures for group A on inferior dental arch (median gr. A 0.42 vs. gr. B 0.28 vs. gr. C 0.25) in all sessions (*p* < 0.01). No differences between pressures for male vs female operators (*p*-value n.s.).

The number of failed intubation attempts (i.e. an operator declaring her/himself to have failed, and/or the attempt lasting more than 30 s even though eventually successful) significantly decreased between the first and the second session (31 vs. 6, *p* < 0.0001).

**Fig. 3:**
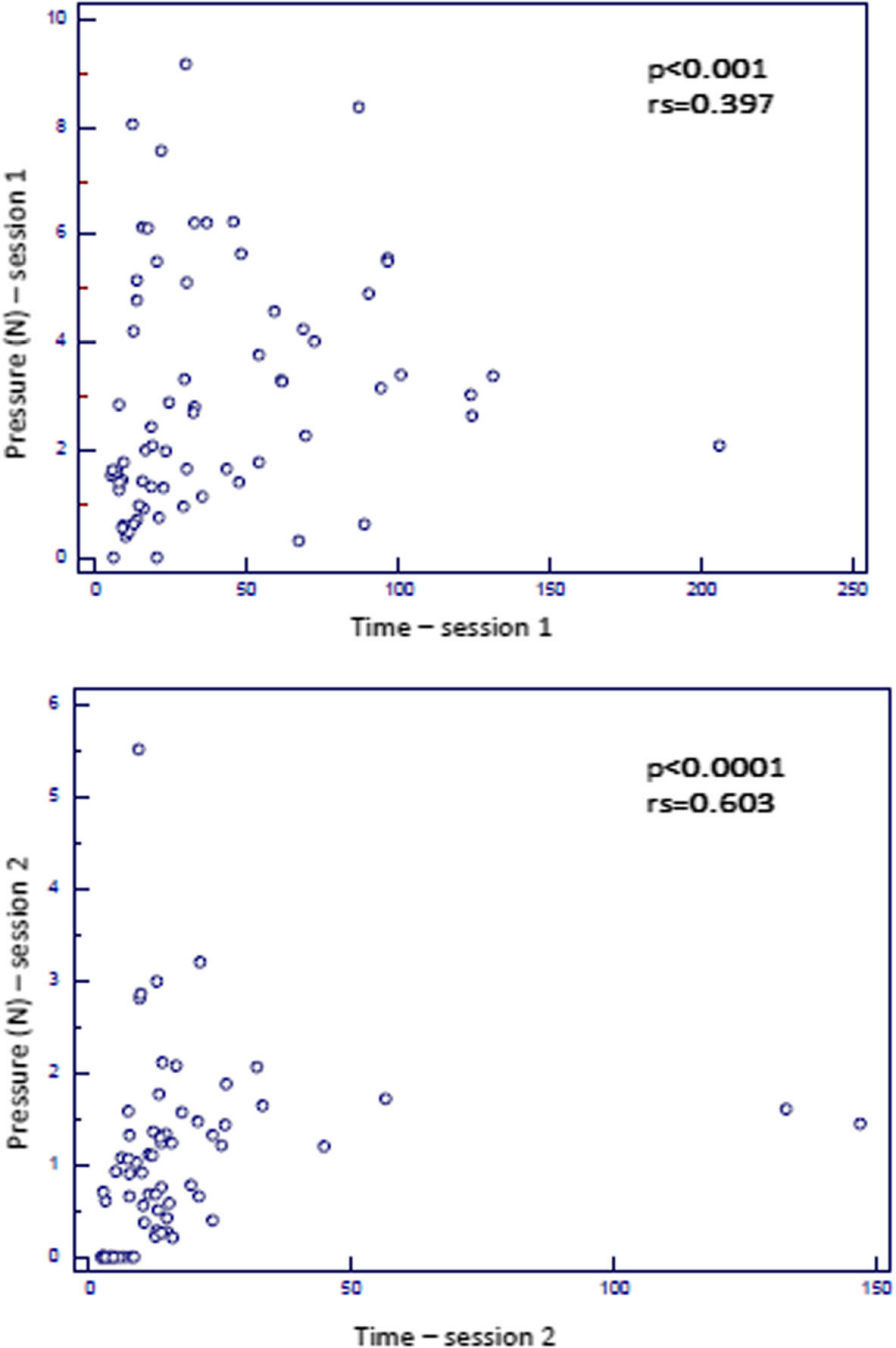
Correlation between time and pressure in first and second session

In all the sessions, procedure time and pressure values were correlated (first session: *p* < 0.001, r_s_ = 0.397; second session: *p* < 0.0001, r_s_ = 0.603 - Fig. 3).

Detailed results of the interview to participants about their satisfaction are reported in Table 1 – part B.

## Discussion

### Skill trainer

At present, to the best of our knowledge, there is no commercial system allowing to judge pressure during intubation training. In our study, we used a skill trainer equipped with force sensors for the retraining of expert medical professionals in intubation procedures. Considering a sample with experience in neonatal intensive care, our aim was to investigate its potential use as an instrument for procedure optimization. We do not exclude its utilization in less expert hands (for example, in pediatric residents), but in our opinion this should follow practice on commercial intubation models, to acquire basic technique. Our novel manikin allowed for accurate feedback of pressures applied, in order to safe soft tissues when operators perform the procedure in vivo. A correct execution of intubations is very important in reducing potential damage on oral and airway structures. This issue is often underestimated in clinical practice, especially in emergencies, where more importance is attributed to a rapid completion of the maneuver. Moreover, we demonstrated operator improvement in terms of time to intubation with reduced applied forces.

In our opinion, offering skill training on a high-difficulty model allows the optimization of the procedure, achieving an automated execution of gentler gestures in easier clinical situations. Moreover, it encourages a deeper reflection on the procedure by the participants. Our opinion about skill trainer is shared by most of our participants, as demonstrated by the results of the second interview.

Moreover, real time feedback of our skill trainer allows a playful approach to the issue, which guarantees a serene and motivated participation in the sessions, according to simulation principles.

We received a good feedback about our skill trainer and its fidelity. Consistently with literature, skill trainer rigidity due to not suitable material represents an issue for the majority of participants, with potential impact on skill trainer fidelity levels and procedure execution. In addition to stiffness related to the commercial model, we believe the allocation of force sensors on external side of the device has a role on this issue. Five operators indicated stiffness as the main problem which forced them to apply greater pressures than in clinical practice. However, the analysis of video-recordings of the attempts demonstrated that these operators strived to obtain a better view of the glottis in order to complete intubations. After debriefing about the difficulties of intubation maneuvers in our skill trainer, we obtained a significant pressure reduction on all critical points. One operator asserted that the applied force level was higher than in clinical practice during the first session, but lower in the second one. After debriefing, the majority of the operators gave a positive feedback about the use of our skill trainer as difficult intubation model, which is consistent with our aim.

In addition to the stiffness of the skill trainer, the method used for attached the sensors to the manikin potentially affected the precision of their location on dental arches. We believe that this issue could underestimate the pressure values recorded in these sites. Anyway, this still proved to be a better solution, if compared with glue as in the previous work [12].

Moreover, we contend that the system accounting for time pacing is inefficient. The majority of participants, especially during the first session, did not pay attention to the 30s and following sound signals, continuing the attempt until success or, eventually, failure. In our opinion, this system lacks fidelity. Its substitution with a more realistic saturation digital monitor, similar to that normally present in NICU, would increase setting fidelity and induce participants to pay increasing attention to decision making and non-technical skills.

We collected opinions about device fidelity as well as any suggestions for potential improvements. Among the latter, the possibility to monitor pressure on the cricoid cartilage, normally considered in clinical practice, would considerably improve glottis view in case of difficult intubation. According to our operators feeling, obtaining a better epiglottis and tongue mobility also needs to be carefully considered. A future perspective of our research could be the realization of a new airway model in which the integration of force sensors ab initio would be envisioned, in order to solve the allocation problems described above. In addition, we should also consider more sophisticated measurement methods, to obtain a continuous force plot on the tissues, rather than punctual force values as in this study (e.g. we need to integrate superficial sensors).

We compared our skill trainer with other devices described in the literature. In a recent study by Doreswamy et al. [14], *Neonatal Intubation Trainer* was employed to evaluate pressure levels accomplished by NICU professionals (included nurses) during intubation procedures. They analyzed forces on the superior dental arch using Prescale Ultra Low, a pressure-sensitive film by Fujifilm. They reported a mean pressure of about 568 kPa, which was considered cause of potential damage. They also recorded mean time of procedure (14.7 s) and mean area under pressure (142mm^2^). These variables were then compared between different health professional categories and different procedure experience (more or less than 2 intubations per year). This study only carried out a comparison between health categories, observing almost no significant differences between groups except for procedure time, which was lower in expert participants. The issue of retraining, however, was not addressed.

To the best of our knowledge, no sensorized skill trainer device for neonatal intubation has been previously described. An active skill trainer for adult intubation *(©Difficult Airway Management Simulator MW11* by *©Kyoto Kagaku)* has been recently released, and is equipped with several kinds of sensors: force sensors on dental arches and tongue (not on epiglottis), position sensors for head and neck, tension sensors for airway. There are actuators which respond to operator’s actions, determining high fidelity. The realization of this device was conducted starting from robotic prototypes, which underwent clinical validation [15]. In a recent study by Nakanishi et al. [16], this device was used to compare different performances by novel physicians with direct laryngoscope and video-laryngoscope. They evidence how video-laryngoscope guarantees a lower pressure on tongue during intubation; on the other hand, in contrast with other studies [17] on adult patients, they find a higher pressure on dental arches during video-laryngoscope intubation, probably due to the lack of clinical experience of their participants.

### Study structure

A familiarization phase is necessary before the beginning of the sessions according to most participants. We did not envision this phase as we aimed to make our settings as similar as possible to routine clinical practice, where a difficult intubation case is an unexpected event which increases failure chances. The surprise effect of such a situation would have been eliminated by a familiarization phase.

Our analysis firstly focused on time and pressure trends. We verified a progressive decrease in procedure time and in pressures on epiglottis and inferior dental arch. In our opinion, these functions can be reasonably considered as learning trends. Our results are in keeping with the impressions of our participants, who noticed a significant performance improvement in the second session. They also gave importance to the debriefing phase, aimed at clarifying our setting and device features. We confirmed again the role of an expert debriefer as learning facilitator in simulation scenarios. Indeed, it is well known that debriefing is a key point in training by simulation, since it substantially contributes to improve trainees’ performances.

The epiglottis sensor was the most stressed during intubation attempts. This data is consistent with gesture features. In our opinion, the attempt of obtaining a better glottis view during intubation explains these findings, considered that most participants declare not to intubate a newborn without a sufficient visualization of the glottis. In this case, debriefing allowed a progressive decrease of pressure values, lowering damage possibility. Active instruction giving by a debriefer may also explain the better correlation between pressure values and decrease of procedure time during the second session.

Although our sample size was limited, we found significant differences among participant groups in our study, related to their years of NICU work experience. In particular, pressures of the younger participants are higher on the inferior arch and lower on the epiglottis if compared with the other groups. This could be explained by a more evident forward traction maneuver executed by the younger personnel, and by the fact that nobody in the younger group loads the epiglottis during intubation attempts.

We believe that the analysis of these differences during debriefing allowed for an effective explanation of intubation gesture. Therefore, our skill trainer could be a valid instrument to achieve an optimization of intubation procedures. Our skill trainer could also represent a starting point for the realization of retraining in intubation procedures for expert medical professionals. Intubation procedure retraining should indeed be carried out as, in its absence, procedural competence regarding airway management has been documented to decrease rapidly [18]. Thus, the maintenance throughout continuing medical education of medical procedural competences has to be emphasized, as strongly as their primary acquisition. In the United States, maintenance programs started in 2000, with the institution of Maintenance of Certification (MOC) by the American Board of Medical Specialties (ABMS). Several studies demonstrated how MOC participants significantly increase selected competences and how simulation is necessary to optimization of clinical practice [19]. In Italy, no similar programs have been officially scheduled so far.

## Conclusions

In this work, we described the technical realization and validation of a novel sensorized skill trainer as a model of difficult intubation for expert operators’ retraining.

We recorded pressure values on critical points (primary outcome) and time of procedure (secondary outcome) during two intubation sessions. Our results evidenced learning trends for both pressure and time in intubation procedure, with statistically significant differences between two sessions for pressure values of epiglottis and inferior dental arch.

We compare our study with other works existing in literature. To the best of our knowledge, no sensorized skill trainer has been previously described and no studies nowadays addressed expert operators’ retraining as main outcome.

Our results underline importance of simulation retraining for technical skills for expert professionals: basing on these techniques, operators can improve gesture of maneuver, pursuing the objective of more efficient and safe intubation. In addition, we confirmed role of debriefing as key point of simulation training, since it permits analysis of simulation actions and correction of mistakes. Therefore, we believe that maintenance of technical skills throughout simulation retraining must be emphasized in Italy, with the institution of simulation education programs, similarly to what already took place in United States with MOC programs.

## Additional files

Additional file 1: Figure S1. *A. epiglottis*’ force sensor allocation on skill trainer. B. ©LabVIEW graphic interface. For force sensors: green - “untouched sensor”, yellow - “under-threshold touched sensor”, red - “over-threshold touched sensor”. For head position: green - sniffing position, red - inappropriate hyperextension. To the red status are associated two different acoustic alarms. (TIFF 468 kb)

Additional file 2: Table S2. Mean times of operators scheduled for attempts in two session. (DOCX 12 kb)

Additional file 3: Table S3. Mean pressure values scheduled for attempts in two session. We considered data with outliers exclusion. (DOCX 13 kb)

## Abbreviations

EI: Endotracheal intubations
GUI: Graphic user-friendly interface
NICU: Neonatal intensive care unit
NRP: Neonatal resuscitation program

## References

[1] Kattwinkel J. Textbook of Neonatal Resuscitation, 6th Edition: 2010 American Academy of Pediatrics and American Heart Association Guidelines for Neonatal Resuscitation. 2011.

[2] Seow WK, Brown JP, Tudehope DI, O'Callaghan M (1984). Developmental defects in the primary dentition of low birth-weight infants: adverse effects of laryngoscopy and prolonged endotracheal intubation. Pediatr Dent.

[3] Angelos GM, Smith DR, Jorgenson R, Sweeney EA (1989). Oral complications associated with neonatal oral tracheal intubation: a critical review. Pediatr Dent.

[4] SIAARTI (2006). Task force: SIAARTI GUIDELINES: recommendations for airway control and difficult airway management in paediatric patients. Minerva Anestesiol.

[5] Cormack RS, Lehane J (1984). Difficult intubation in obstetrics. Anaesthesia.

[6] Fletcher G, Flin R, McGeorge P, Glavin R, Maran N, Patey R (2004). Rating non-technical skills: developing a behavioural marker system for use in anaesthesia. Cogn Tech Work.

[7] Gaba DM, Howard SK, Fish KJ, Smith BE, Sowb YA (2001). Simulation-based training in anesthesia crisis resource management (ACRM): a decade of experience. Simulation & Gaming.

[8] Halamek LP, Kaegi DM, Gaba DM, Sowb YA, Smith BC, Smith BE (2000). Time for a new paradigm in pediatric medical education: teaching neonatal resuscitation in a simulated delivery room environment. Pediatrics.

[9] Bishop MJ, Harrington RM, Tencer AF (1992). Force applied during tracheal intubation. Anesth Analg.

[10] Hall RS, Desmoulin GT, Milner TE (2008). A technique for conditioning and calibrating force-sensing resistors for repeatable and reliable measurement of compressive force. J Biomech.

[11] FSR Integration guide. Interlink Electronics Website. https://cdn2.hubspot.net/hubfs/3899023/Interlinkelectronics%20November2017/Docs/Datasheet_FSR.pdf. Accessed 8 Feb 2016.

[12] Tognarelli S, Baldoli I, Scaramuzzo RT, Ciantelli M, Cecchi F, Gentile M (2014). Development and validation of a sensorized neonatal intubation skill trainer for simulation based education enhancement. Int J Med Res Health Sci.

[13] Wyckoff MH, Aziz K, Escobedo MB, Kapadia VS, Kattwinkel J, Perlman JM (2015). Neonatal resuscitation 2015 guidelines update for cardiopulmonary resuscitation and emergency cardiovascular care (reprint). Circulation.

[14] Doreswamy SM, Almannaei K, Fusch C, Shivananda S (2015). Compression force on the upper jaw during neonatal intubation: mannequin study. J Paediatr Child Health.

[15] Noh Y, Wang C, Tokumoto M, Jorge S, Ishii H, Takanishi A, Shoji S. Development of Airway Management training system WKA-4: Provide useful feedback of trainee performance to trainee during Airway Management. In 2012 ICME International Conference on Complex Medical Engineering, CME 2012 Proceedings. pp. 423–428. [6275605]. 10.1109/ICCME.2012.6275605.

[16] Nakanishi T, Shiga T, Homma Y, Koyama Y, Goto T (2016). Comparison of the force applied on oral structures during intubation attempts by novice physicians between the Macintosh direct laryngoscope, airway scope and C-MAC PM: a high-fidelity simulator-based study. BMJ Open.

[17] Lee RA, van Zundert AA, Maassen RL (2012). Forces applied to the maxillary incisors by video laryngoscopes and the Macintosh laryngoscope. Acta Anaesthesiol Scand.

[18] Youngquist ST, Henderson DP, Gausche-Hill M, Goodrich SM, Poore PD, Lewis RJ (2008). Paramedic self-efficacy and skill retention in pediatric airway management. Acad Emerg Med.

[19] Steadman RH, Huang YM, Cooper JB (2015). Practice improvements based on participation in simulation for the maintenance of certification in anesthesiology program. Anesthesiology.

