# Supplementary figures and images for: Technical realization of a sensorized neonatal intubation skill trainer for operators’ retraining and a pilot study for its validation
Source: Ital J Pediatr. 2018 Jan 4;44:4. doi: 10.1186/s13052-017-0435-z (PMC5755336; doi:10.1186/s13052-017-0435-z)

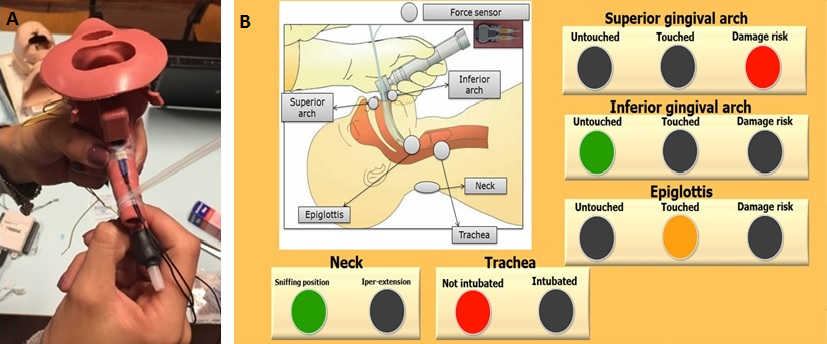

Supplement: Supplementary file 1 — A. epiglottis’ force sensor allocation on skill trainer. B. ©LabVIEW graphic interface. For force sensors: green - “untouched sensor”, yellow - “under-threshold touched sensor”, red - “over-threshold touched sensor”. For head position: green - sniffing position, red - inappropriate hyperextension. To the red status are associated two different acoustic alarms. (TIFF 468 kb) [file 13052_2017_435_MOESM1_ESM.tif]
