# Supplementary material for: Technical realization of a sensorized neonatal intubation skill trainer for operators’ retraining and a pilot study for its validation
Source: Ital J Pediatr. 2018 Jan 4;44:4. doi: 10.1186/s13052-017-0435-z (PMC5755336; doi:10.1186/s13052-017-0435-z)
Supplement: Supplementary file 2 — Mean times of operators scheduled for attempts in two session. (DOCX 12 kb) [file 13052_2017_435_MOESM2_ESM.docx]

Table S2. **Mean times of operators scheduled for attempts in two session.**

|  | | Operator mean times (s) | | |
| --- | --- | --- | --- | --- |
|  |  | I SESS | II SESS | II SESS (outliers) |
| Attempts | 1 | 58,38±50,99 | 21,4±32,91 | 22,13±35,62 |
|  | 2 | 47,84±37,62 | 14,68±11,38 | 13,3±8,3 |
|  | 3 | 37,61±29,87 | 14,51±12,89 | 12,09±5,44 |
|  | 4 | 25,88±31,7 | 19,28±37,22 | 10,68±8,06 |
|  | 5 | 32±35,07 | 9,39±6,14 | 9,59±6,57 |
