# Supplementary material for: Technical realization of a sensorized neonatal intubation skill trainer for operators’ retraining and a pilot study for its validation
Source: Ital J Pediatr. 2018 Jan 4;44:4. doi: 10.1186/s13052-017-0435-z (PMC5755336; doi:10.1186/s13052-017-0435-z)
Supplement: Supplementary file 3 — Mean pressure values scheduled for attempts in two session. We considered data with outliers exclusion. (DOCX 13 kb) [file 13052_2017_435_MOESM3_ESM.docx]

Table S3. **Mean pressure values scheduled for attempts in two session**. We considered data with outliers exclusion.

|  | | Mean pressure values (N) | | | | | | | | |
| --- | --- | --- | --- | --- | --- | --- | --- | --- | --- | --- |
|  |  | **EPIGLOTTIS** | | | **SUPERIOR ARCH** | | | **INFERIOR ARCH** | | |
|  |  | I SESS | II SESS | II SESS (outliers) | I SESS | I SESS (outliers) | II SESS | I SESS | I SESS (outliers) | II SESS |
| Attempts | 1 | 2,98±2,59 | 1,22±1,01 | 1,07±0,87 | 0,23±0,27 | 0,25±0,28 | 0,02±0,08 | 0,44±0,45 | 0,44±0,47 | 0,18±0,16 |
|  | 2 | 3,07±1,93 | 0,94±0,58 | 0,96±0,6 | 0,75±2,05 | 0,21±0,22 | 0,04±0,1 | 0,34±0,14 | 0,33±0,15 | 0,15±0,14 |
|  | 3 | 3,17±2,61 | 0,61±0,62 | 0,64±0,63 | 0,18±0,23 | 0,17±0,24 | 0,02±0,09 | 0,37±0,33 | 0,30±0,18 | 0,18±0,18 |
|  | 4 | 2,82±2,19 | 1,24±1,51 | 0,91±0,92 | 0,21±0,25 | 0,23±0,25 | 0,05±0,17 | 0,26±0,17 | 0,25±0,17 | 0,17±0,16 |
|  | 5 | 2,82±1,99 | 0,91±0,83 | 0,82±0,79 | 0,19±0,26 | 0,19±0,27 | 0,03±0,12 | 0,23±0,16 | 0,23±0,16 | 0,16±0,13 |
